# Maternal Care and Offspring Development Trajectories in Mice: Effects of Alzheimer’s Disease Genotype and Maternal Age

**DOI:** 10.1002/alz.095724

**Published:** 2025-01-09

**Authors:** Alesia V Prakapenka, Sophia Parpala, Mallica Cary, Edith Ramirez, Stacy Kujawa, Andrew Hawkey

**Affiliations:** ^1^ Midwestern University, Downers Grove, IL USA

## Abstract

**Background:**

Pregnancy and birth uniquely alter female physiology, biology, and behavior. Contrasting findings on pregnancy and AD risk association suggest that confounding variables (e.g., age at birth) mediate pregnancy’s role in AD risk. We aim to elucidate the impact of age at first birth on female behavioral and brain health at middle‐age in the triple‐transgenic AD mouse model. Since strain and genotype differences in maternal care and offspring development exist, maternal care and offspring development trajectories as a function of genotype and maternal age were assessed.

**Methods:**

Wildtype (WT; C57BL/6J) and AD virgin females were paired with WT males for pregnancy and birth to occur at 2‐m‐o. Gestational weight gain and litter characteristics (i.e., pup number, sex ratio, body weight) were recorded. Maternal care measures included retrieval of four pups to nest following 5‐min separation on postnatal day 2 (PND2) and evaluation of maternal activities for two hours on PND3 and PND17. Offspring development was assessed by righting reflex (PND5‐8) and negative geotaxis (PND10‐13) tests.

**Results:**

All mice were pregnant and gave birth, with two WT litters lost after birth. AD dams tended to have increased gestational weight gain compared to WT dams [p < 0.1]. Gestational weight gain was similar across genotypes after accounting for pup number and lost litters. All dams successfully retrieved pups on the retrieval task. Genotype by pup interaction [F_(3, 36)_ = 2.814, p = 0.05] indicated that latency to retrieve increased with each subsequent pup in AD versus WT dams. Preliminary analyses suggest AD dams spent more time digging during the task compared to WT dams, which may modulate observed pup retrieval differences. Dam genotype impacted offspring development trajectories, whereby WT litters exhibited longer righting reflex latencies and shorter negative geotaxis latencies compared to AD litters.

**Conclusions:**

Findings suggest that maternal care and offspring development trajectories differ as a function of AD genotype in 2‐m‐o dams. Ongoing study evaluates 5‐6‐m‐o and 8‐9‐m‐o maternal ages. Future work will assess age at first birth effects on maternal health outcomes (e.g., behavior, neuropathology) in these dams at middle‐age, correlating to observed maternal care and offspring development measures.

